# When gratitude and cooperation between friends affect inter-brain connectivity for EEG

**DOI:** 10.1186/s12868-020-00563-7

**Published:** 2020-04-07

**Authors:** Michela Balconi, Giulia Fronda, Maria Elide Vanutelli

**Affiliations:** 1 Department of Psychology, Catholic University of Milan, Milan, Italy; 2 Research Unit in Affective and Social Neuroscience, Catholic University of Milan, Milan, Italy; 3 Department of Philosophy, University of Milan, Milan, Italy

**Keywords:** Gratitude, Emotions, EEG, Inter-brain connectivity, Cooperation, DLPFC

## Abstract

**Background:**

Recently several studies in the psychological and social field have investigated the social function of gift exchange as a useful way for the consolidation of interpersonal and social relationships and the implementation of prosocial behaviors. Specifically, the present research wanted to explore if gift exchange, increased emotional sharing, gratitude and interpersonal cooperation, leading to an improvement in cognitive and behavioral performance. In this regard, neural connectivity and cognitive performance of 14 pairs of friends were recorded during the development of a joint attention task that involved a gift exchange at the beginning or halfway through the task. The moment of gift exchange was randomized within the pairs: for seven couples, it happened at task beginning, for the remaining seven later. Individuals’ simultaneous brain activity was recorded through the use of two electroencephalograms (EEG) systems that were used in hyperscanning.

**Results:**

The results showed that after gift exchange there was an improvement in behavioral performance in terms of accuracy. For what concerns EEG, instead, an increase of delta and theta activation was observed in the dorsolateral prefrontal cortex (DLPFC) when gift exchange occurred at the beginning of the task. Furthermore, an increase in neural connectivity for delta and theta bands was observed.

**Conclusion:**

The present research provides a significant contribution to the exploration of the factors contributing to the strengthening of social bonds, increasing cooperation, gratitude and prosocial behavior.

## Background

Recent studies have investigated the act of giving or receiving a gift as a specific and crucial moment of interpersonal exchange that can influence the development of social relationships and prosocial behavior [1, 2]. In detail, the emotional processes underlying this moment have been one of the main focus of previous research as a possible trigger for the construction of significant bonds. The gift exchange, indeed, produces some specific effects that contribute to strengthening the relationship between individuals, as firstly an increase of emotional contagion and emotional sharing caused by the sense of reciprocity perceived by inter-agents [3]. Furthermore, the gift exchange, enhancing trust, affective consideration and mutual solidarity, increases the consolidation of deeper and cohesive relationship [4, 5], creating a type of mutual obligation that leads individuals to develop a sort of implicit pact [6, 7]. Furthermore, bilateral beneficial exchanges, such as gift exchange, have been shown to increase the sense of gratitude experienced by individuals, strengthening the construction of joint actions that create a sense of interdependence [4, 8–13]. Specifically, gratitude has been consistently explored during gift exchange, since it carries a positive social value, increasing prosociality and subjective wellbeing [14, 15]. In particular, it has been observed that individuals, after gift receiving, are pushed through the reward systems to repay the benefit obtained, positively influencing their performance [16–19].

Indeed, gratitude is caused by the perception of what is identified as a genuine effort by the receiver [20] and is associated with beneficial effects in social relationships [14, 21–24] and with the implementation of prosocial behavior [21, 25].

Moreover, gift exchange, developing solid and functional interpersonal relationships, enhance individuals’ cooperative attitudes by increasing the sense and the perception of social recognition and enhancing a collective comparison [26]. Cooperation, indeed, can reinforce the sense of social inclusion and interpersonal cohesion thanks to the implementation of joint actions [27, 28]. The implementation of coordinated actions required by cooperative behavior led neurosciencentist to consider inter-agent actors as a new, unique and complex system that cannot be studied as the result of the sum of the two parts.

Indeed, the neural responses of such a dyad underlie the pursuit of a common goal [29]. The social link and the brain synchronization between the two members of the couple can be justified by the possibility of sharing positive feelings with another person, both acting and responding emotionally [30, 31]. Indeed, previous research underlined that emotions are the basis of prosocial behavior, influencing individuals’ choice to cooperate or compete with someone [32, 33] and mediating interpersonal and social relationships [34, 35].

To explore these mechanisms new and advanced methodologies have been developed. In this regard, an innovative research paradigm of cognitive and social neuroscience has been successfully proposed, that is hyperscanning [36, 37]. It allows the simultaneous acquisition of the neurophysiological responses of two participants who interact naturally during a joint task [38].

Hyperscanning can be performed with various neuroscientific techniques with a double apparatus.

However, compared to the use of other neurophysiological measures, such as functional magnetic resonance imaging (fMRI) or functional near-infrared spectroscopy (fNIRS), the use of EEG-based hyperscanning allows us to obtain a better temporal resolution and to record the interactions of the two inter-agents moment by moment [39].

Several previous studies have measured the brain synchrony between two individuals with the use of EEG during different coordinated interaction. For example, Astolfi and colleagues [40] proposed participants a game to be solved together, consisting of moving a rolling ball towards a specific region of the screen with a virtual bar. In another case, participants played a computerized version of the table tennis [41] by using competing or cooperating strategies. In this last case, interbrain synchrony was significantly higher. Moreover, a study of Balconi [42] showed a systematic response within the prefrontal regions (PFC) after receiving a positive feedback assessing a good performance and a winning situation.

Considering specific functions of cerebral areas, indeed, the prefrontal regions consistently emerged as pivotal in coordinated joint actions [43]. Interestingly, such brain networks proved to take a role also concerning emotional processes, affective consideration and gratitude effects [34, 44, 45].

However, although previous studies have already explored the potential of using hyperscanning in some social processes [39, 46], no one has ever explicitly focused on the mechanisms underlying gift experience in creating cooperative behaviors.

Thus, in the present study, we aimed at investigating EEG functional connectivity and synchronicity between two brains during a joint performance involving gift exchange. In detail, we thought that emotional sharing, the mutual solidarity and formation of cohesion relationships experienced during the gift exchange could increase cooperation, improving individual’s behavioral performance. Thus, we performed a joint paradigm consisting of an attentive task in which participants, coupled in dyads, had to cooperate. The behavioral and cortical responses of the two members of the couple were recorded by means of an EEG-based hyperscanning technique.

Specifically, the primary objective of the present study was to investigate if and how the behavioral performance and the brain activity of the participants improved after the gift exchange.

Secondly, we wanted to explore whether the specific moment of gift donation (beginning or halfway through the task) could be useful in modifying subjects’ brain and behavioral responses.

Indeed, two different experimental conditions were created to be compared. In one case, gift exchange occurred at the beginning of the task, while in the other case the offer took place halfway through the task. Thirdly, we aimed at observing which brain regions could be more involved in the implementation of cooperative behavior.

We hypothesized that emotional sharing, mutual solidarity and gratitude experienced during gift exchange could reinforce bond formation and cooperative behaviors both at a behavioral and neural level, with improved behavioral performance, synchronizing the individuals’ responses in terms of accuracy, and enhanced neural activity, increasing the neural connectivity between the two individuals of the dyad. Indeed, as demonstrated by previous studies, an improvement of individuals behavioral responses occurred in the presence of a greater interpersonal bond [28, 37, 47, 48].

Moreover, we expected that such effect was more pronounced during the first condition, which involved gift donation at the beginning of the task. We believe that an earlier exchange could better reinforce the interpersonal bond. Indeed, it was shown that the gift exchange as a prosocial behavior may changes the relationships in a positive manner, increasing the development of cohesive relationships, shared emotion, sense of reciprocity [3] and attuning [5], that may gradually induce an increase in our sense of cooperation, enforcing our positive emotions, the sense of social inclusion and interpersonal cohesion [34, 35], with higher level at the end of the interaction.

Finally, we expected that a specific neural network involving the frontal regions could be activated after gift exchange due to the involvement of emotional and empathic processes [34, 49–55]. In particular, we hypothesized to observe greater brain responsiveness of frontal delta and theta activity, which are more involved in affective and emotional sharing processes [56–59]. Specifically, we expected to observe an increase of frontal delta activity due to the involvement of this frequency band in emotional engagement [60]. Furthermore, we expected to observe an increase of frontal theta activity after gift exchange considering the involvement of this frequency band in empathic, social processes [56, 57] and strategic control in social context [61, 62]. Finally, considering that during the gift exchange a greater sharing of emotional responses [3] occurs, which strengthens the bond and the implementation of cooperative behaviors between individuals [31, 63], we expected to see a more considerable increase of delta and theta inter-brain connectivity in both the donor and the receiver after the beginning gift exchange.

## Method

### Participants

For the experiment, fourteen pairs of friends’ participants of the same sex were involved (M = 23.07; SD = 1.05). Specifically, the couples had to be formed by members involved in a consolidated friendship relationship, who attended and saw each other regularly. Participants provided their written consent to participate in the research. Subjects with normal or correct-normal visual acuity were recruited, while subjects presented with a pathological neurological or psychiatric history were excluded. The research was conducted following the Helsinki Declaration and was approved by the local ethics committee of the Department of Psychology of the Catholic University of Milan.

### Procedure

The participants were placed side by side at 60 cm from two computers divided by a black screen to prevent eye contact, avoiding the possibility for the two members of the dyads to look or talk to each other. Specifically, they were given a common task that required a gift to be exchanged, asking one member of each dyad (donor) to donate a gift to the partner (receiver) at the beginning or halfway through the task. In particular, the donor was asked to give the gift to the receiver face to face. The choice of gift exchange was suggested by the experimenter and can consist in accessories, objects and tickets for visiting a museum or a concert.

**Fig. 1:**
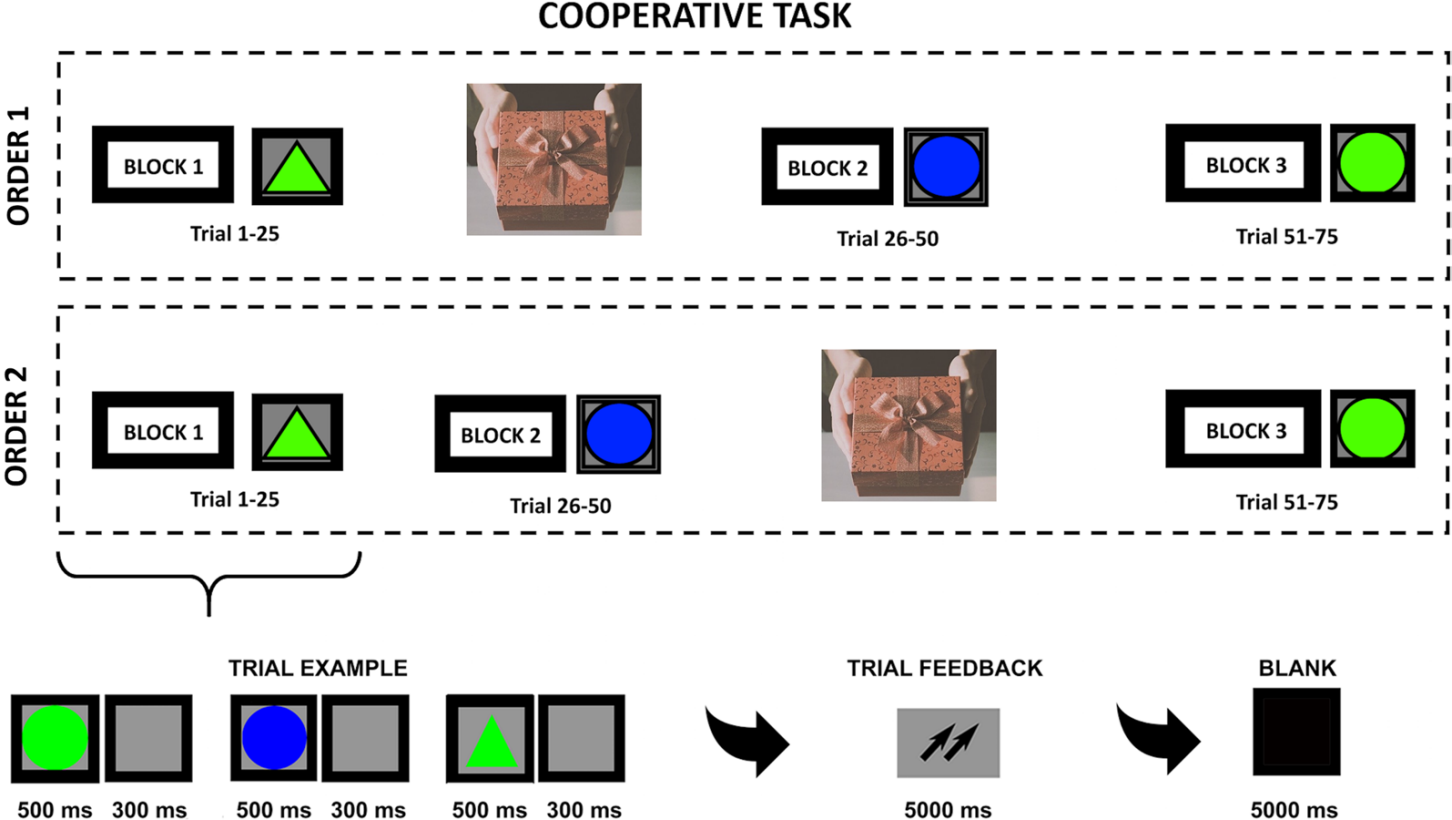
The figure shows the experimental procedure. Two different procedures for the cooperative task were performed. The first, order 1, is divided in: block 1 (a control condition), gift exchange (image retrieved from pixabay), block 2 and block 3. The second, order 2, is divided in: block 1, block 2, gift exchange (image retrieved from pixabay), and block 3. Each block was composed by 25 trials and after each trial members of the couple received a feedback on their cooperation level

The gift exchange occurred randomized for seven couples before the beginning of the first part (after block 1); for the other seven pairs at the end of the second block. Based on this, two different procedures were used: the first (order 1) included block 1 (a baseline condition), gifts exchange and blocks 2 and 3; the second (order 2) included the following composition: block 1 (a baseline condition), block 2, gifts exchange and block 3 (Fig. 1).

Specifically, in blocks 1, 2 and 3, participants were asked to carry out a cooperative task modified by a previous computerized version [37, 51] consisting in the execution of a selective attention task that required participants to synchronize their responses in terms of accuracy (ACC) and reaction time (RTs).

Specifically, participants were required to memorize a target, that could be a triangle or a circle of green or blue color, which was later to be recognized among other stimuli proposed by pressing the left/right keys of the computer keyboard.

Specifically, each block was composed by the presentation of 25 trials. The structure of each trial was the following: the presentation of the stimulus for 500 ms (msec), the presentation of an inter-stimulation interval (ISI) of 300 ms and the presentation of an interval between trials (ITI) of 5000 ms duration. Moreover, after the presentation of three stimuli, participants received a feedback of their cooperation’s degree, consisting in two upward arrows, use to indicate a good level of cooperation, in two down arrows, use to indicate a low level of cooperation, and in a dash, use to indicate a mean performance.

In this previous version of the task, cooperative nature of participants was stressed with the execution of four task blocks with a manipulated a priori good feedback on their cooperation’s level and four task blocks with a manipulated a priori negative feedback on their cooperation’s level.

In the present version of the task, the same task was administered to participants asking them to synchronize the accuracy and the speed of their responses. In this version, differently from the previously, participants were asked to execute only three blocks, composed by 25 trials, before and after a gift exchange.

Also in this version, participants after the execution of three stimuli received a feedback on their cooperation’s level, consisting in two upward arrows or a dash. The aim of the feedback was, therefore, to allow participants to implicitly learn the use of good cooperative strategies and also to synchronize their responses to that of their workmates. After the task was carried out, both members of the couple were given a questionnaire to explore the perception of the partner and torque tuning level during the entire task. Specifically, the questionnaire asked the following questions: “What was the perception of your workmate in the first phase of the game?”, “What was the perception of your workmate in the second phase of the game?”, “What was the perception of your collaboration and degree of gratitude in the first phase of the game?”, “What was the perception of your collaboration and degree of gratitude in the second phase of the game?”. The members of the couples responded to these items by assigning a score on the Likert scale from 1 (perception of non-synchrony/non-cooperation) to 3 (perception of great harmony and cooperation). Three expert judges coded the answers of all the participants.

### EEG recording and analysis

For the recording of EEG signal, two 12-channel EEG systems (V-AMP: Brain Products, München) were used by placing the electrodes in AFF1h, Fz, AFF2h, FFC3h, FFC4h, C3, Cz, C4, P3, Pz, P4 positions. Two ElectroCap electrode with Ag/AgCl electrodes were used to record EEG.

Specifically, data acquisition took place with a sampling frequency of 500 Hz, a frequency band of 0.01–40 Hz and an impedance below 5 kΩ. After visually evaluating the signal, ocular, muscle, and movement artifacts were rejected after data segmentation by visual inspection. Baseline and condition-specific average power spectra were computed starting from artifact-free segments (Fast Fourier Transform: resolution = 0.5 Hz; periodic Hanning window). The EEG data were subsequently filtered in the past band in the frequency band: delta (0.5–4 Hz), theta (4–8 Hz), alpha (8–12 Hz), beta (14–20 Hz). For each EEG channel (Ch), a calculation of the average individual power value was made for each experimental condition. Before pre-gift training condition, after the 120-s baseline record, subjects were given a familiarization task.

## Data analysis

A preliminary analysis was conducted for the questionnaire response.

Successively three sets of analyses were performed with respect to behavioral (ACC; RTs) and neurophysiological (EEG measures) dependent measures. The following paragraphs specifically described the analyses we applied to two behavioral measures and to two neurophysiological measures, respectively the single brain analyses and successively the inter-brain connectivity analyses. See the following section for each of them.

### Questionnaire responses

Two mixed-model ANOVAs were applied to dyadic tuning scoring and perceived cooperation with Block (pre vs. post) as within-subjects factor, and Condition (Cond: order 1 vs order 2) and Role (Role: donor vs. receiver) as between-subjects factors.

### Behavioral analyses

By using E-prime Software, ACC and RTs were obtained for each subject during baseline and the successive two blocks. ACC was calculated as the percentage of correct responses on the total responses, while RTs were computed starting from stimulus presentation. Then, two mixed-model ANOVAs were applied to ACC and RTs with Block (1 baseline vs 2 vs 3) as within-subjects factor, and Condition (Cond: order 1 vs order 2) and Role (Role: donor vs. receiver) as between-subjects factors. For all the ANOVA tests, the degrees of freedom were corrected using Greenhouse–Geisser epsilon when appropriate. Post hoc comparisons (contrast analyses) were applied to the data. A Bonferroni test was applied for multiple comparisons. In addition, the normality of the data distribution was preliminary tested (kurtosis and asymmetry tests). The normality assumption of the distribution was supported by these preliminary tests.

### EEG analysis: single-brain analyses

For single-brain analyses, the mean EEG power for each channel and each frequency band was calculated by averaging data within each block. The effect size in every block was calculated for each channel and subject as the difference of the means of the block (m1 or m2) and the baseline divided by the standard deviation (sd) of the baseline, respectively:

$${\text{d1}} = {{\left( {{\text{m1}} - {\text{baseline}}} \right)} \mathord{\left/ {\vphantom {{\left( {{\text{m1}} - {\text{baseline}}} \right)} {\text{sd}}}} \right. \kern-0pt} {\text{sd}}};$$

$${\text{d2 }} = {{\left( {{\text{m2}} - {\text{baseline}}} \right)} \mathord{\left/ {\vphantom {{\left( {{\text{m2}} - {\text{baseline}}} \right)} {{\text{sd }}\left( {{\text{Cohen}}'{\text{s d value}}} \right)}}} \right. \kern-0pt} {{\text{sd }}\left( {{\text{Cohen}}'{\text{s d value}}} \right)}}.$$

Then, 4 different regions of interest (ROIs) were calculated by averaging left/right homologous channels: the values obtained from Ch1 and Ch3 were averaged as representative of the activity of superior frontal gyrus (SFG, Brodmann Area 6) [64], from Ch4 and Ch5 were averaged as representative of the activity of the dorsolateral prefontal cortex (DLPFC, Broadmann area 46) [65], from Ch6 and Ch8 were averaged as representative of the activity of the dorsal premotor cortex (DPMC, Brodmann Area 4) [66] and from Ch9 and Ch11 were averaged as representative of the activity of primary motor cortex (PMC, Brodmann Area 3) [67]. Subsequently, one mixed-model ANOVAs was applied to such indices with Condition (Cond, order 1 vs order 2), Block (3) and ROI (DLPFC, SFG, DPMC, PMC) used as within-subjects factors, and Role (2) as between-subjects factors.

### EEG analysis: inter-brain connectivity analyses

A third step was performed to calculate inter-subjects correlational indices finalized to compute the synchronization within each dyad for EEG measure. Correlational indices were applied to the same time interval adopted for EEG single brain analysis (i.e. stimulus time duration then averaged for each block and each channel). For the inter-brain connectivity calculation, the partial correlation coefficient Πij was computed to obtain functional connectivity indices. They were obtained by normalizing the inverse of the covariance matrix Γ = Σ^−1^:

$$\begin{aligned} \Gamma \, = \, \left( {\Gamma {\text{ ij}}} \right) \, = \, \Sigma^{-1}{\text{ inverse of the covariance matrix}} \hfill \\ \Pi ij=\frac{-\Gamma ij}{\sqrt{\Gamma ii\Gamma jj}} {\text{ partial correlation matrix}} \hfill \\ \end{aligned}$$

It quantifies the relationship between two signals (i, j) independently from the other [68]. Cross-channel calculation was adopted to obtain the covariance matrix.

Such indices (r values) were successively entered as variables into mixed-model ANOVA tests, applied to EEG measures, with Cond, Block and ROI as within-subjects factors, and Role as between-subjects factors.

## Results

### Behavioral and questionnaire data

For ACC measurement, ANOVA revealed a significant effect for Cond (F [1, 27] = 9.08; p < 0.001; η2 = 0.32), with a better performance (higher percentages) for order 1 than order 2; Block (F [2, 28] = 9.12; p < 0.001; η2 = 0.31) and Cond * Block (F [2, 53] = 9.13; p < 0.001; η2 = 0.30).

**Fig. 2:**
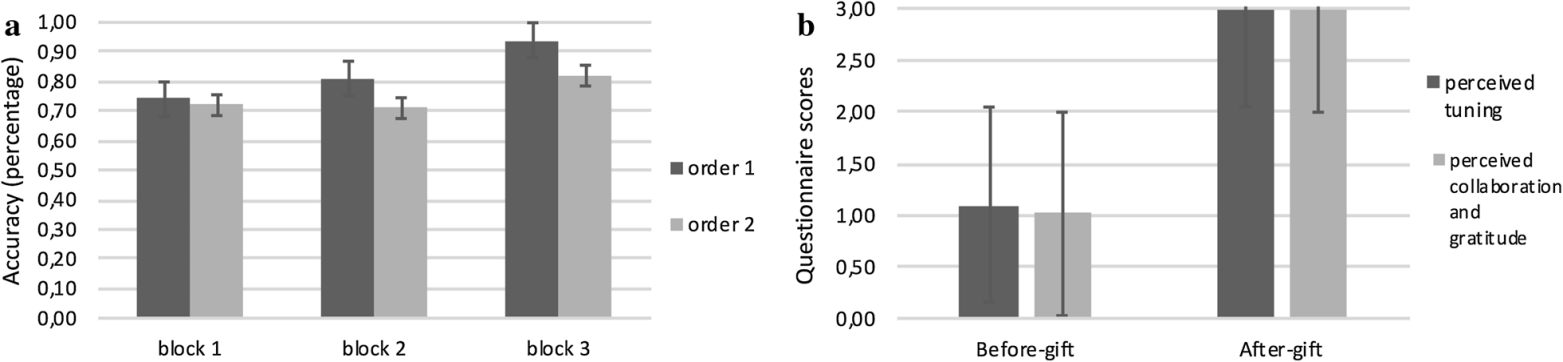
**a** The figure shows the percentage of performance accuracy (ACC) for order 1 and order 2 for block 1, block 2 and block 3. **b** The figure shows the questionnaire responses about perceived tuning and perceived collaboration and gratitude before and after gift exchange

Specifically, as shown by post hoc comparison applied to interaction effects, order 1 revealed higher ACC in block 2 more than block 1 (F [1, 27] = 8.90; p < 0.001; η2 = 0.29) and in block 3 more than block 1 (F [1, 27] = 11.09; p < 0.001; η2 = 0.34). In addition block 2 differed from block 3 (F [1, 27] = 8.54; p < 0.001; η2 = 0.29). In contrast, order 2 showed higher ACC in block 3 more than block 1 (F [1, 27] = 11.12; p < 0.001; η2 = 0.35) and block 2 (F [1, 27] = 9.65; p < 0.001; η2 = 0.32) (Fig. 2a). For RTs, no effect was statistically significant.

For what concerns questionnaire responses, for dyadic tuning, ANOVA revealed a significant effect for Block (F [1, 27] = 23.56; p < 0.0001; η2 = 0.92), with higher perceived tuning after (M = 2.99; SD = 0.05) than before (M = 1.10; SD = 0.04) gift exchange. With regard to perceived collaboration and gratitude, ANOVA revealed a significant effect for Block (F [1, 27] = 23.33; p < 0.0001; η2 = 0.91), with higher perceived cooperation and gratitude after (M = 2.98; SD = 0.04) than before (M = 1.01; SD = 0.05) gift exchange (Fig. 2b).

### Single-brain analyses

The statistical analyses were applied to d1 and d2 dependent measures for each frequency band.

**Fig. 3:**
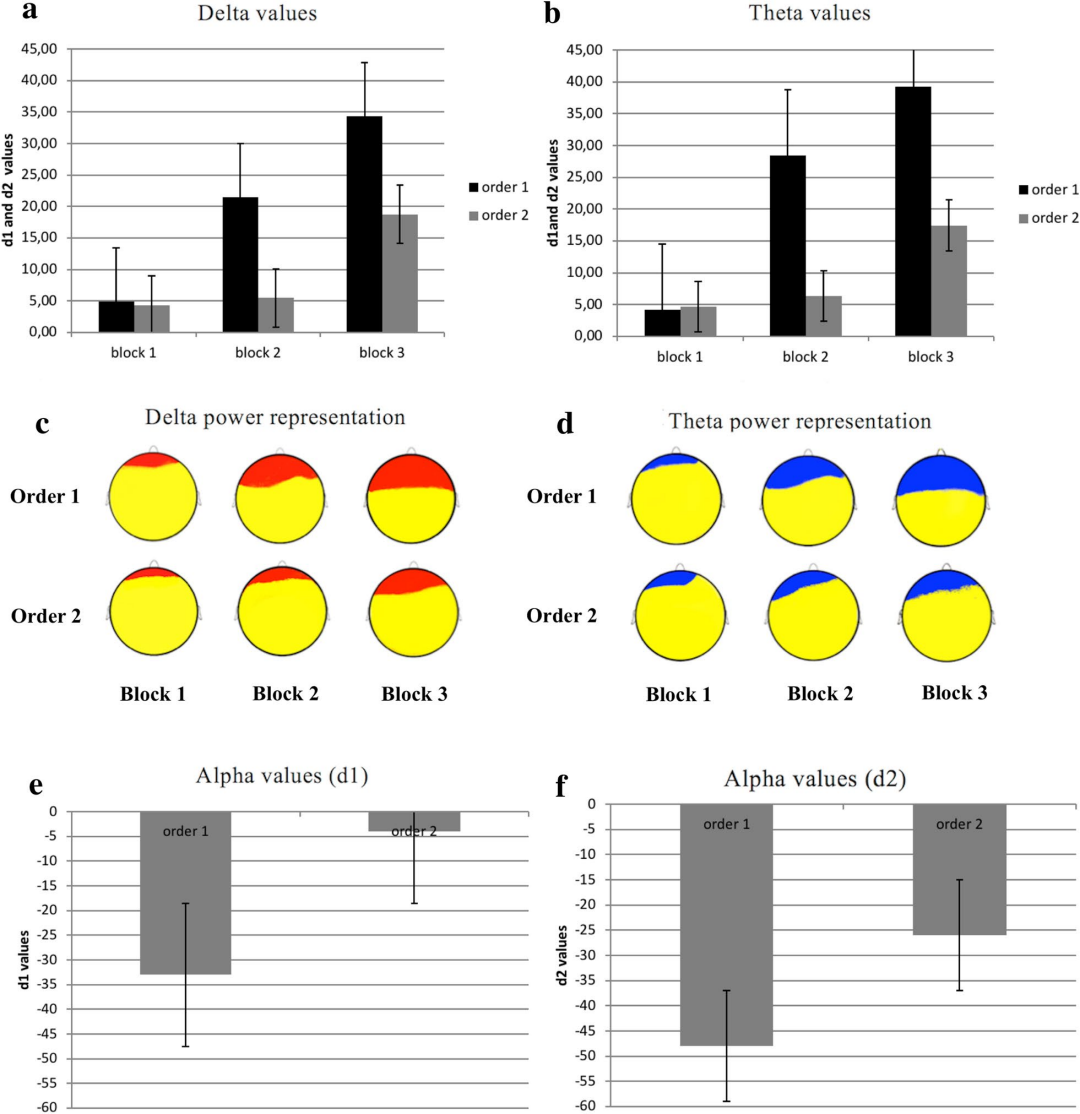
**a** Histograms of delta values (d1 and d2) in DLPFC area for order 1 and order2. **b** Histograms of theta values (d1 and d2 values) in DLPFC area for order 1 and order 2. **c** Delta power representation (d1 and d2 values) for order 1 and 2. The red color represents the areas in which compare an increase of delta d1 and d2 values. **d** Theta power representation (d1 and d2 values) for order 1 and 2. The figure shows an increase of theta in DLPFC area for order 1 than order 2. The blue color represents the areas in which compare an increase of theta d1 and d2 values. **e** Histogram of alpha values (d1 values) in DLPFC area for order 1 and order 2. **f** Histogram of alpha values (d2 values) in DLPFC area for order 1 and order 2

About delta, as shown by ANOVA, Cond * Block * ROI interaction effect was significant (F [6, 70] = 7.90, p < 0.01, η2 = 0.29). Specifically, as revealed by post hoc comparisons, there was an increase of delta (for both d1 and d2 values) in DLPFC area for order 1 than order 2 (respectively F [1, 27] = 8.77, p < 0.01, η2 = 0.29; F [1, 27] = 7.32, p < 0.01, η2 = 0.27) (Fig. 3a, c). In addition in order 1, d1 and d2 differed from each other, with higher DLPFC activity in block 3 than block 2 (higher d2 than d1 values) (F [1, 27] = 8.10, p < 0.01, η2 = 0.30). Similarly, in order 2, d2 was higher than d1 in the DLPFC (F [1, 27] = 7.98, p < 0.01, η2 = 0.27).

About theta, Cond * Block * ROI interaction effect was significant (F [6, 70] = 7.12, p < 0.01, η2 = 0.29). Specifically, as revealed by post hoc comparisons, there was an increase of theta (increased d1 and d2 values) in DLPFC area for order 1 than order 2 (respectively F [1, 27] = 9.09, p < 0.01, η2 = 0.32; F [1, 27] = 8.92, p < 0.01, η2 = 0.29) (Fig. 3b, d). In addition, in order 1, d1 and d2 differed from each other, with higher d2 than d1 values (F [1, 27] = 8.55, p < 0.01, η2 = 0.30). Similarly, in order 2, d2 was higher than d1 in the DLPFC (F [1, 27] = 8.02, p < 0.01, η2 = 0.29).

Finally, about alpha, Cond * Block interaction effect was significant (F [6, 70] = 6.55, p < 0.01, η2 = 0.25). Indeed apha decreased in order 1 more than order 2 (decreased d1 and d2, respectively F [1, 27] = 8.35, p < 0.01, η2 = 0.30; F [1, 27] = 6.98, p < 0.01, η2 = 0.26) (Fig. 3e, f). For beta no significant effect was found.

### Inter-brain connectivity analyses

**Fig. 4:**
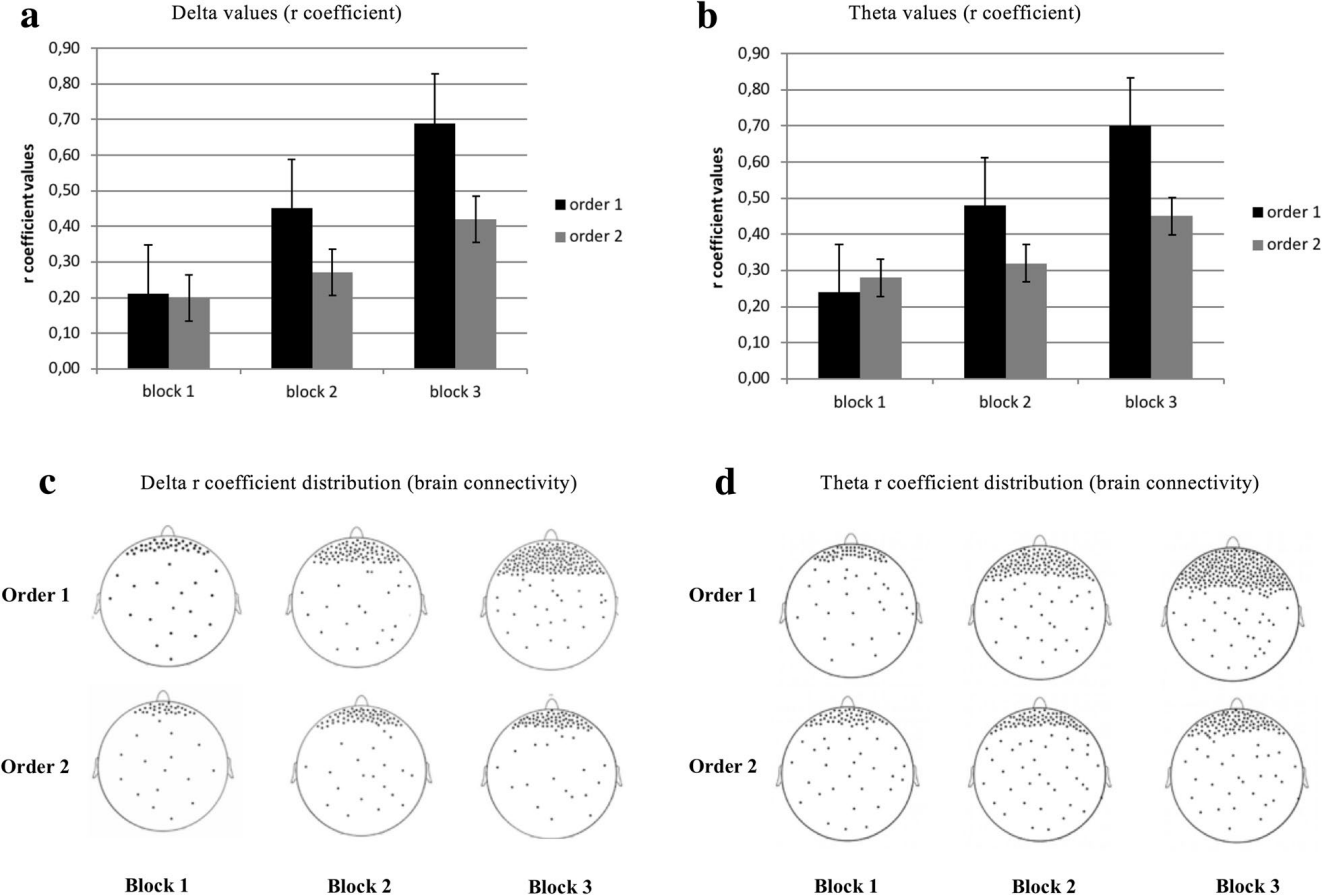
**a** Histograms of delta values (r coefficient) in DLPFC area for order 1 and order 2. **b** Histograms of theta values (r coefficient) in DLPFC area for order 1 and order 2. **c** Graphical representation of delta R coefficient distribution (brain connectivity), represented by dots within the cortical maps. Specifically, the figure shows an increase of delta brain connectivity in DLPFC area for order 1 and 2. **d** Graphical representation of theta R coefficient distribution (brain connectivity), represented by dots within the cortical maps. Specifically, the figure shows an increase of theta brain connectivity in DLPFC area for order 1 and 2

For delta band, as shown by the ANOVA, Cond * Block * ROI interaction effect was significant (F [6, 54] = 7.16, p < 0.01, η2 = 0.29). Specifically, pairwise post hoc comparisons revealed significant higher connectivity in DLPFC (higher r values) for order 1 more than order 2 in block 2 and block 3 (respectively F [1, 13] = 9.11, p < 0.01, η2 = 0.32; F [1, 13] = 8.44, p < 0.01, η2 = 0.30) (Fig. 4a, c). In addition higher connectivity was observed in order 1 for block 3 more than block 2 (F [1, 13] = 6.34, p < 0.01, η2 = 0.26).

For theta band, as shown by the ANOVA, Cond * Block * ROI interaction effect was significant (F [6, 54] = 8.09, p < 0.01, η2 = 0.30). Specifically, pairwise post hoc comparisons revealed significant higher connectivity in DLPFC for order 1 more than order 2 in block 2 and block 3 (respectively F [1, 13] = 8.34, p < 0.01, η2 = 0.30; F [1, 13] = 8.79, p < 0.01, η2 = 0.30) (Fig. 4b, d). In addition higher connectivity was observed in order 1 for block 3 more than block 2 (F [1, 14] = 7.65, p < 0.01, η2 = 0.27).

## Discussion

The aim of the present study was to explore the relationship between experience of gift exchange and cooperation. Specifically, we wanted to observe how sharing a pleasant moment, such as gift donation, could increase cooperation and have beneficial effects on individuals’ behavioral performance and brain activity and synchrony. In this regard, an attentive task was proposed that involved a gift exchange at the beginning or halfway through the task. The analyses revealed some significant results: (1) The gift exchange provoked an improvement of subjects’ behavioral responses in terms of accuracy; (2) The moment in which the gift is donated is associated with specific behavioral and neural effect (activation of individual frequency bands); (3) The act of giving always produces positive effects regardless of the moment in which the gift is exchanged; (4) The gift exchange results in the recruitment of a specific brain network involving the DLPFC; (5) When the gift is exchanged before the task is performed, there is a greater inter-brain tuning and connectivity in the delta and theta activity, especially in the DLPFC; (6) There is no significant difference regarding the role (donor or receiver) by the two members of the dyads.

Beginning with the first significant result (1), an improvement in behavioral performance was observed in terms of better accuracy, after the gift exchange. As already reported by previous studies, indeed, subjects behavioral responses improve in the presence of a greater interpersonal bond [28, 37, 47, 48]. The improvement in behavioral performance may be explained with increased cooperative strategies between individuals [69]. One another important effect we have revealed may be associated to the increased sense of gratitude which is spied out following the cooperative conditions required by the task. Indeed, it has been observed that the sense of gratitude developed within “economic” relationships, such as the gift exchange, leads individuals to experience a sense of trust and adaptive gratitude, as demonstrated by behavioral economics games in which individuals avoid logics related to personal enrichment in order to reward partners for a sense of gratitude experienced by the perception of cooperative behavior [70], which leads them to strengthen cohesion and collaboration. The increase of cooperation and sense of gratitude perceived by the couples’ members is also confirmed by the results of the questionnaire administered at the end of the task which reported an increase in perceived tuning and perceived collaboration and gratitude after the gift exchange. However, about the second main result, such effect, as well as neural responses, was mediated by the specific timing when the gift was exchanged. In detail, if the gift was donated at the beginning of the interaction between the members of the couple, the effect was stronger. This finding can be explained by defining the gift as an input for the strengthening and maintenance of the cooperative link between the inter-acting individuals, as evidenced by the detection of more accurate responses in order 1 than in order 2.

Indeed, gift exchange can be the premise for the construction of a significant bond implying commitment and positive emotions. Previous research underlined that, when cooperating, a significant emotional tuning emerge besides behavioral synchronization and cognitive tuning [48, 71].

A similar result was also found in neural activity. Specifically, greater activation of delta and theta bands was observed following gift exchange in order 1, compared to order 2, in the DLPFC.

This result may be because positive emotions experienced during gift exchange plays an essential role in the construction of cooperative bonds that immediately influence social interaction, creating a strong bond and a greater emotional harmony among individuals [72–74]. Indeed, as demonstrated by previous studies [60, 75], theta and delta modulations were considered as specific markers of motivational and emotional components of the subjects’ engagement. In particular, greater activation in the delta band can be attributed to the fact that the latter represents a significant indicator of emotional behavior, cognition and social skills [56, 76]. Moreover, delta activity results to be involved in motivational systems and emotional salience detection [60]. Similarly, greater activation of theta band may be due to involvement in social processes, as demonstrated by some research that have observed a greater theta activation during empathic processes [56, 57]. Indeed, as demonstrated by different studies [61, 62], theta activity is involved in strategic control and attentive significance of emotional situations [77, 78].

Thus, we can suppose that the positive emotions associated with gifts donation provided at the beginning of the interpersonal exchange function as a social glue thanks to reciprocity mechanisms, activating those frequency bands that are more involved in emotional processing [60, 77, 79].

Furthermore, following earlier gift exchange (order 1), a decrease in alpha activity was observed. Such result reveals how, besides emotional attuning, the cooperative bond also induced more synchronized cognitive-related processes. In fact, alpha decrease can be interpreted as an increase in cognitive activation [79–81].

Concerning the fourth main result, it is possible to observe how these neural effects occurred in a specific brain area, the DLPFC. As already demonstrated by previous studies [50, 51, 82], indeed, this region seems to be related to social and interpersonal relations regulation, cooperative behavior acts and empathic processes. In fact, empathy allows sharing the mental representations of two inter-acting individuals, resulting in an improvement of cognitive abilities and strategies [83, 84].

Concerning the fifth result, greater inter-brain synchronization (increased r values) was observed for delta and theta bands in the DLPFC in order 1 compared to order 2. Specifically, in order 1, an increase in connectivity in block 3 compared to block 2 emerged. These results showed once again, and consistently with our hypothesis, how the anticipated gift exchange increases the connectivity and the synchronization between the members of the couples. These results are also confirmed by participants’ subjective responses revealing higher perceived attunement and cooperation following gift exchange. As shown by previous research, indeed, sharing a pleasant experience increases the feeling of being part of a whole and the sense of interpersonal cohesion, leading individuals to perceive themselves more in tune with each other [28, 51, 85]. The increased connectivity between two individuals, indeed, involves the transition from an interpersonal perspective to an inter-agent representation. Specifically, when the neural connectivity between two people increases, we can assume the adoption of common strategies and modification of self-goals, directed towards the creation of synergistic actions, and the understanding of the actions of others [37], that increase brain-to-brain coupling. The improvement of individuals attunement enhances individuals’ attentive and behavioral synchronization [85, 86], giving a somatosensory structure that facilitates the intentions and actions understanding [87–89]. In particular, in this case the increase of delta and theta inter-brain connectivity after the beginning gift exchange can be due to the fact that the sharing of positive emotions induced by gift exchange improve the social link and the brain synchronization between individuals [30, 31].

Finally, it was noted that the role played by the two members of the dyads was irrelevant. In the light of this evidence, it can, therefore, be said that both the act of giving and that of receiving a gift produce an improvement and a strengthening of cooperative ties. This result could underline, as demonstrated by previous studies [90], that the implementation of a prosocial behavior can represent a social reward, even without a material return.

## Conclusion

In conclusion, the present study shows how consequent positive emotions caused by the gift exchange strengthen the construction and implementation of a cooperative link between inter-acting individuals, increasing interpersonal and social bonding. The positive effects of increased cooperation between individuals after gift exchange appear to be visible both at a behavioral level and at a neural level. Some points to examine in future studies could be: to compare and examine different interpersonal and social ties, to explore possible differences in cooperative style concerning gender (man-woman) and to explore how other personal factors of individuals can influence the increase in behavior cooperative. Furthermore, in future studies, the exploration of individuals’ perception of cooperation and gratitude could be collected immediately after gift exchange to avoid that participants have to recall their feeling at the end of the task. Moreover, in future studies could also be considered the possible effects of different gift’s valence, for example more neutral or negative. Finally, future research could include adjunctive measures for the assessment of personality traits of individuals (such as empathic traits) in order to see how much they influence behavioral performance and the level of cooperation of individuals.

## Data Availability

The datasets used and/or analysed during the current study are available from the corresponding author on reasonable request.

## Abbreviations

EEG: Electroencephalograms
DLPFC: Dorsolateral prefrontal cortex
fMRI: Functional magnetic resonance imaging
fNIRS: Functional near-infrared spectroscopy
PFC: Prefrontal cortex
ACC: Accuracy
RTs: Reaction time
SFG: Superior frontal gyrus
DLPFC: Dorsolateral prefontal cortex
DPMC: Dorsal premotor cortex
PMC: Primary motor cortex
ROIs: Regions of interest
ISI: Inter-stimulation interval
msec: Milliseconds
ITI: Inter-trial Interval
Hz: Hertz
Ch: Channel
Cond: Condition
sd: Standard deviation
